# Selective Omission of Ureteral Access Sheath in Retrograde Intrarenal Surgery: Surgical and Safety Outcomes from a Single-Center Retrospective Cohort Study

**DOI:** 10.3390/jcm15062345

**Published:** 2026-03-19

**Authors:** Po-Sung Liang, Yu-Jun Chang, Jian-Kai Chen, Hung-Jen Shih

**Affiliations:** 1 Divisions of Urology, Department of Surgery, Changhua Christian Hospital, Changhua 500209, Taiwan; 184963@cch.org.tw (P.-S.L.); 720610@cch.org.tw (J.-K.C.); 2 Big Data and Digital Promotion Center, Changhua Christian Hospital, Changhua 500209, Taiwan; 83686@cch.org.tw; 3 Division of Urology, Department of Surgery, Tungs’ Taichung MetroHarbor Hospital, Taichung 435403, Taiwan; 4 Department of Post-Baccalaureate Medicine, College of Medicine, National Chung Hsing University, Taichung 402202, Taiwan; 5 Department of Urology, School of Medicine, College of Medicine, Taipei Medical University, Taipei 110301, Taiwan; 6 Department of Urology, Yuan Rung Hospital, Changhua 510005, Taiwan

**Keywords:** retrograde intrarenal surgery, ureteral access sheath

## Abstract

**Introduction:** The ureteral access sheath (UAS) is commonly used in retrograde intrarenal surgery (RIRS) to improve vision, lower intrarenal pressure (IRP), and facilitate access. However, concerns regarding ureteral injury remain. We conducted this study to evaluate the surgical efficacy and safety of a selective omission strategy for UAS use during RIRS in patients with small renal stones (<10 mm) or in cases where UAS placement is technically difficult. **Materials and Methods:** This retrospective study included consecutive patients who underwent single-surgeon RIRS at Changhua Christian Hospital between October 2020 and April 2023 for renal or upper ureteral stones. Sheathless RIRS was performed in patients with stones < 10 mm, or in whom insertion of a 10/12 Fr UAS was unsuccessful despite successful advancement of an 8 Fr semirigid ureteroscope, and when the surgeon estimated the procedure could be completed within 2 h. All procedures used a holmium laser with a 9 Fr or 7.5 Fr flexible ureteroscope. No patients were pre-stented, and all received postoperative double-J stenting. **Results:** Among 55 patients, 18 (32.7%) underwent sheathless RIRS and 37 (67.3%) underwent UAS-assisted RIRS. Stone size was significantly smaller in the sheathless group (12 mm vs. 17 mm, *p* = 0.001). The 3-month stone-free rate (SFR) was 66.7% in the sheathless group and 62.2% in the UAS group (*p* = 0.745). Operative time was similar between groups (77 vs. 85 min, *p* = 0.154), with no statistically significant differences in postoperative pain or length of hospital stay. In the UAS-assisted group, six patients developed febrile urinary tract infection, of whom two progressed to sepsis; all recovered after antibiotic therapy. No fever or sepsis occurred in the sheathless group. On multivariable analysis, lower calyceal stone location was independently associated with SFR, whereas UAS use was not. **Conclusions:** In a selected cohort (stones < 10 mm or difficult UAS insertion with an expected operative time < 2 h), sheathless RIRS was feasible and showed no statistically significant differences in SFR or perioperative outcomes compared with UAS-assisted RIRS. However, due to selection bias, stone-size imbalance, and limited statistical power, these findings should not be interpreted as procedural equivalence and require confirmation in adequately powered studies with stratified/adjusted analyses.

## 1. Introduction

The use of a ureteral access sheath (UAS) is considered an important adjunct in retrograde intrarenal surgery (RIRS), as it may help reduce intrarenal pressure (IRP), improve endoscopic visibility, and facilitate repeated access to the renal pelvicalyceal system [1]. However, ureteral safety concerns related to UAS use have been reported. Studies have documented ureteral wall damage following UAS insertion as assessed using standardized grading systems [2]. Subjectively difficult UAS placement and prolonged placement time have been associated with high-grade ureteral wall injury [3]. Furthermore, one study documented tissue ischemia and subsequent alterations due to reperfusion following UAS insertion [4]. Ureteral stricture development has also been documented as a postoperative complication [5]. In contrast, other reports have found no significant association between UAS use and the development of abnormal postoperative upper tract imaging [6]. A systematic review further concluded that, based on the current evidence, a direct relationship between UAS use and ureteral injury cannot be established, and patients selected for UAS may even appear to have a lower risk of ureteral injury [7]. Consequently, the routine use of UAS remains controversial.

In daily practice, concern about potential ureteral injury continues to discourage many urologists from using UAS. A growing corpus of literature indicates that sheathless RIRS may be feasible for selected patients, with several authors advocating for the omission of a sheath either intentionally or when UAS insertion is infeasible. Mahmood et al. prospectively assessed a sheathless and fluoroscopy-free technique utilizing holmium laser dusting mode, documenting elevated stone-free rates (SFRs) with predominantly minor complications, and emphasized sheath avoidance as a potential method to mitigate sheath-related issues [8]. Surgeons may avoid UAS in practical scenarios due to challenges such as difficult insertion resulting from a constricted ureter, concern for ureteral injury, and the intention to reduce instrumentation, especially when the expected stone burden is small and a dusting approach is intended. Concurrent advancements in flexible ureteroscope technology—characterized by reduced caliber and enhanced image quality—may further facilitate endoscopic navigation and visualization. Simultaneously, the extensive implementation of holmium:YAG laser lithotripsy has facilitated effective fragmentation and dusting techniques, potentially diminishing the necessity for repeated access for basket extraction in specific cases [9].

Against this background, we conducted this study to assess the safety and efficacy of sheathless RIRS in certain patients, particularly those with small stone burdens or in whom UAS placement was difficult, and in whom the anticipated operative time was less than 2 h.

## 2. Materials and Methods

### 2.1. Study Design and Patient Selection

We conducted a retrospective study from October 2020 to April 2023 at Changhua Christian Hospital. All consecutive patients who underwent RIRS for renal or upper ureteral stones by a single surgeon (HJ Shih) were included in the analysis. Inclusion criteria were: (1) adult patients (≥18 years old) with renal or upper ureteral stones, (2) those with stones amenable to endoscopic management, and (3) patients undergoing primary RIRS without prior ureteral stenting. Exclusion criteria included patients with (1) anatomical abnormalities of the urinary tract (e.g., horseshoe kidney, ureteropelvic junction obstruction), (2) active urinary tract infection at the time of surgery, (3) severe bleeding disorders, and (4) incomplete medical records or imaging follow-up.

### 2.2. UAS Selection Strategy

The rationale for the selective omission of the UAS was grounded in practical intraoperative feasibility and the characteristics associated with the stones. In our practice, UAS installation was originally attempted in all cases. Sheathless RIRS was conducted solely when at least one of the following criteria was satisfied: (1) stone burden < 10 mm, rendering repeated instrument exchanges unnecessary, or (2) unsuccessful insertion of a 10/12 Fr UAS despite the successful advancement of an 8 Fr semirigid ureteroscope, indicating sufficient ureteral compliance without necessitating forceful dilation, with an estimated operative time of less than 2 h based on the surgeon’s judgment. This selective strategy demonstrates a realistic, safety-focused approach rather than a desire to entirely avoid UAS utilization. The decision-making process remained consistent throughout the trial duration and was conducted by a single skilled surgeon, thus reducing inter-operator variability.

### 2.3. Lithotripsy Protocol

Throughout the study period, two different flexible ureteroscopes were utilized based on institutional availability. Between October 2020 and January 2022, a single-use 9 Fr flexible ureteroscope (HU30, HugeMed, Shenzhen HugeMed Medical Technical Development Co., Ltd., Shenzhen, China) was utilized. From January 2022, a single-use 7.5 Fr flexible ureteroscope (HU30S, HugeMed, Shenzhen HugeMed Medical Technical Development Co., Ltd., Shenzhen, China) was implemented for all RIRS procedures. All surgeries employed a flexible ureteroscope without prior ureteral stenting. After initial cystoscopy and identification of the ureteral orifice, an 8-French semirigid ureteroscope was advanced into the ureteral orifice to assess ureteral patency and to dilate the ureter gradually. A 0.035-inch hydrophilic guidewire was then placed under semirigid ureteroscopic guidance. In the UAS group, a 10/12-French UAS was inserted under fluoroscopic guidance. The flexible ureteroscope was then passed through the UAS for stone management. In the sheathless group, following guidewire placement, the flexible ureteroscope was subsequently introduced over the guidewire for intrarenal access. Lithotripsy was conducted via a holmium:YAG laser system, namely the Cyber Ho 100 (100 W, Quanta System, Samarate, Italy). During the procedure, stones were initially disintegrated utilizing a combination of fragmentation and dusting techniques to attain sufficient size reduction. Subsequently, a popcorn mode was employed to further pulverize the residual pieces into small particles, promoting spontaneous clearance (Fragmentation mode: 1.8 J, 20 Hz; dusting mode: 1.5 J, 20 Hz; popcorn mode: 1.2 J, 30 Hz). Intraoperative irrigation was delivered using a pressure bag set at 100 mmHg, ensuring that the maximum irrigation pressure remained below 100 mmHg. The operating surgeon adjusted irrigation flow intraoperatively according to endoscopic visibility. In the sheathless group, careful attention was paid to irrigation fluid balance, with regular deflation of the collecting system achieved by temporarily withdrawing the ureteroscope to allow drainage. Surgical endpoints included complete stone fragmentation, achieving adequate stone-free status, or when the operative time exceeded 120 min.

### 2.4. Postoperative Management and Follow-Up

Subsequent to RIRS, all patients underwent insertion of a double-J (DJ) stent for postoperative drainage and prophylaxis for ureteral obstruction caused by ureteral edema. The DJ stent was removed between postoperative days 14 and 20. The patients received follow-up image at 3 months postoperatively to assess stone-free status, which was defined as residual stone fragments less than 4 mm on either non-contrast computed tomography (CT) or kidney–ureter–bladder (KUB) radiography combined with renal ultrasound. The choice of imaging modality was based on patient preference and clinical indication. Early postoperative septic complications were defined as those occurring within 30 days following RIRS. Fever is defined as an elevation in body temperature of ≥38 °C. Sepsis is characterized as “a life-threatening organ dysfunction resulting from a dysregulated host response to infection” and evaluated by the fast Sequential Organ Failure Assessment (q-SOFA) score [10].

### 2.5. Data Collection and Outcome Measures

The patient and perioperative variables incorporated into the multivariable logistic regression model were chosen based on clinical significance and existing research, including age, sex, body height, body weight, body mass index (BMI), stone size, stone position, postoperative visual analogue scale (VAS), urethral pain, flank pain, hospital stays, operation time, and stone-free status. The primary outcome was the SFR at 3 months. Secondary outcomes included operative time, perioperative pain, and length of hospital stay.

### 2.6. Statistical Analysis

Continuous variables are reported as medians and interquartile ranges (25th–75th percentiles), whereas categorical variables are expressed as counts and percentages. The Mann–Whitney U test was employed to compare median continuous variables, whilst univariable analysis of categorical variables was conducted using Fisher’s exact test or Chi-square tests. Univariable and multivariable logistic regression analyses were utilized to determine characteristics independently correlated with the SFR. Variables included in the multivariable model were selected based on clinical relevance. Adjusted odds ratios (ORs) with 95% confidence intervals (CIs) were calculated to identify independent predictors of stone-free status. A two-sided *p*-value of less than 0.05 was deemed statistically significant.

## 3. Results

### 3.1. Patient Characteristics

Patient demographics and baseline characteristics are detailed in Table 1. A total of 55 patients were included, of whom 18 (32.7%) underwent sheathless RIRS, and 37 (67.3%) received UAS-assisted RIRS. Baseline demographics—including age, sex distribution, BMI, and stone location—did not differ significantly (all *p* > 0.05).

Notably, stone size differed significantly between the two groups. Patients receiving UAS exhibited a greater median stone size than those in the sheathless cohort (17 mm vs. 12 mm, *p* = 0.001).

### 3.2. Postoperative Outcomes, with Versus Without UAS Use

Table 2 displays perioperative results and postoperative recovery parameters. The median operative time was not significantly different between the two groups (77 min for the sheathless group versus 85 min for the UAS group, *p* = 0.154). Postoperative pain evaluation indicated no substantial changes in early postoperative VAS values. The incidence of urethral and flank pain was low, with no statistically significant difference between groups (both *p* > 0.999). Length of hospital stay was short in both groups (median, 1 day) and did not differ significantly (*p* = 0.663). Stone-free status at 3 months was attained in 12 patients (66.7%) in the sheathless group and 23 patients (62.2%) in the UAS-assisted group, with no statistically significant difference noted (*p* = 0.745).

No clinically apparent ureteral injury or severe hematuria was observed intraoperatively. Post-operative complications were recorded according to the Clavien–Dindo system till 14 days after the operation. Most of the complications were Grade I, including flank pain and mild gross hematuria. In the UAS-assisted group, six patients developed urinary tract infections with fever (Grade II), of whom two subsequently progressed to sepsis (Grade IV). All patients recovered after antibiotic therapy. No fever or sepsis was observed in the sheathless group.

### 3.3. Factors Associated with Stone-Free Rate

Parameters correlated with stone-free status are presented in Table 3. In univariable logistic regression analysis, lower calyceal stone location was associated with lower odds of achieving stone-free status (OR 0.167, 95% CI 0.033–0.834, *p* = 0.029). The association retained statistical significance following adjustment for clinically pertinent factors in the multivariable model (adjusted OR 0.172, 95% CI 0.032–0.923, *p* = 0.040). UAS use was not significantly associated with stone-free status in either univariable or multivariable analysis. Stone size, age, sex, BMI, and scope caliber were also not significantly associated with stone-free status.

## 4. Discussion

This retrospective study demonstrates that sheathless RIRS may be a feasible alternative to conventional UAS-assisted RIRS in appropriately selected patients. Despite being performed mainly in patients with smaller stone burdens or difficult UAS insertion with an anticipated operative time of 2 h or less, sheathless RIRS achieves no significantly different SFRs, operational duration, and postoperative results compared to those performed with UAS. These findings support consideration of a selective, individualized approach to UAS use during RIRS rather than routine application. Our results are consistent with an emerging paradigm in endourology that emphasizes tailoring strategies to stone characteristics and patient-specific factors instead of default utilization of all available devices.

Despite the prevalent use of UAS to diminish IRP by enhancing irrigation outflow, providing multiple access, and protecting the flexible ureteroscope [11], apprehensions remain about ureteral wall ischemia, mucosal damage, and the potential for long-term stricture development. The literature presents conflicting evidence regarding the benefits and risks of routine UAS use. Prior research indicates that the utilization of UAS does not inherently result in clinically significant ureteral damage but protects the kidney [12], and Venkatachalapathy et al. developed a novel technique called “Rule of five” to perform ureteral dilatation smoothly before UAS placement [13]. However, the notion of sheathless RIRS has garnered significant attention in recent research, especially with regard to the reduction of ureteral damage and the customization of UAS use for specific patients. A recent large multicenter retrospective analysis indicated that the use of UAS during RIRS was correlated with a similar SFR but higher perioperative morbidity [14]. Recently, a systematic review and meta-analysis based on 12,993 patients from 22 studies showed that the utilization of UAS did not markedly influence SFR, complications, or duration of hospitalization. Nonetheless, it extended both operative and fluoroscopy duration. Routine utilization of UAS is not endorsed, and determinations should be tailored to individual patients [15]. De Coninck et al. presented a comprehensive analysis of UAS application, asserting that future practices should transition from routine usage to selected implementation based on stone burden, ureteral anatomy, and technical progress [16]. Nonetheless, the majority of current discussions are either theoretical or predominantly concentrate on device-related outcomes instead of clinical efficacy. Currently, the absence of consensus persists in shaping clinical practices. Our research provides clinical evidence endorsing this paradigm by showing that the selective omission of UAS does not undermine SFRs or surgical safety in meticulously selected individuals. However, though our data add clinical evidence in a real-world setting, safety in terms of ureteral injury should be interpreted cautiously because ureteral injury was not graded using a standardized intraoperative system, and long-term stricture surveillance was limited. The similar postoperative pain profiles and complication rates indicate that sheathless RIRS does not elevate ureteral morbidity. In contrast to previous findings that generally compare UAS and non-UAS techniques, our cohort particularly represents real-world situations in which UAS insertion is technically difficult or clinically unwarranted due to minimal stone burden. The decision to use UAS should be based on stone complexity, anticipated operative time, and technical feasibility rather than as the default approach. This approach may help avoid unnecessary ureteral instrumentation while maintaining short-term outcomes in selected patients.

The regression analysis revealed that lower calyceal stones, rather than the use of UAS, were independently correlated with a reduced probability of attaining stone-free status. This corresponds with existing studies emphasizing lower pole anatomy and gravity-dependent positioning as critical factors influencing remaining fragments after RIRS [17]. Lower pole stones present unique challenges due to acute infundibulopelvic angle, long infundibular length, and narrow infundibular width. Those factors impede the spontaneous passage of stone fragments, regardless of whether UAS is used. The gravitational disadvantage of the lower pole makes complete stone clearance more difficult. The absence of correlation between UAS utilization and SFR further supports the hypothesis that anatomical considerations may exert a more significant influence on surgical outcomes than the choice of equipment.

The sheathless approach offers several potential advantages. First, it avoids the mechanical trauma associated with UAS insertion, which requires significant ureteral dilation and may cause mucosal injury even with careful technique. Second, it eliminates the continuous radial compression of ureteral blood vessels that occurs during prolonged UAS placement, potentially reducing ischemic injury. Third, it may shorten operative time by eliminating the time required for sequential dilation and sheath placement. In our series, although operative time was not significantly shorter in the sheathless group (77 vs. 85 min, *p* = 0.154), this may reflect the smaller stone size in that group and the surgeon’s deliberate efforts to ensure adequate drainage between periods of irrigation. The ability to safely manage these stones without UAS may reduce procedural costs (eliminating the cost of UAS and sequential dilators), minimize radiation exposure (less fluoroscopy time for sheath placement), and potentially reduce ureteral injury [14]. Potential reductions in device-related costs and fluoroscopy use for sheath placement are theoretical in this context; however, procedural cost and radiation exposure were not collected in this study, and ureteral injury was not systematically graded. Therefore, these potential advantages should be interpreted as speculative rather than demonstrated outcomes in our cohort.

The De Coninck et al. comprehensive review on UAS use highlighted several important considerations for future practice patterns. Ongoing technological progress could broaden the applications for sheathless RIRS. The advent of smaller-caliber digital flexible ureteroscopes, enhanced deflection mechanisms, intraoperative IRP monitoring devices, and high-power laser systems—such as thulium fiber laser (TFL)—has been reported to improve stone disintegration efficiency and may reduce the need for repeated scope exchanges and potentially lower irrigation requirements. Because these technologies were not evaluated and IRP was not measured in the present study, their impact on sheathless RIRS cannot be inferred from our data. Nevertheless, these advances may support future investigations examining whether sheathless RIRS can be safely extended to patients with moderately sized stones or more complex anatomy [16]. As laser and ureteroscope technologies continue to evolve, the threshold stone size for sheathless RIRS may expand beyond 10 mm in future practice; however, prospective studies are required to confirm feasibility and safety.

This study has significant limitations. First, this study is a retrospective methodology. There is an absence of objective ureteral injury assessment and adequate follow-up time to evaluate long-term complications. Future prospective studies with larger cohorts, direct intraoperative grading of ureteral injury, and radiographic follow-up will be crucial to confirm the long-term safety of sheathless RIRS, especially for potential subclinical ureteral injury. Second, all procedures were conducted by a single surgeon, thereby minimizing variability in technique and decision-making. Third, the sheathless group was intentionally selected for patients with smaller stones (especially <1 cm) or difficult UAS insertion when the procedure was judged feasible within 2 h, which also led to a significant stone-size imbalance between groups. Therefore, the comparable SFR should be interpreted as showing non-inferior outcomes of sheathless RIRS only in this selected population, rather than procedural equivalence for all RIRS patients. Further studies with stone-size-stratified/adjusted analyses and prospective validation are needed to determine the broader applicability of sheathless RIRS. Fourth, although no sepsis occurred in the sheathless group, the safety of sheathless RIRS cannot be definitively established from this small study. In addition, IRP was not measured, which further limits safety assessment. Larger cohort studies with IRP monitoring are needed to better evaluate the safety of sheathless RIRS. Fifth, the small sample size limits the statistical power of this study and increases the risk of type II error. Therefore, the absence of statistically significant differences should not be interpreted as proof of equivalence, and our findings should be considered preliminary. The results should be interpreted cautiously without overextending conclusions regarding procedural equivalence. Larger studies with adequate sample-size calculations are needed to validate the non-inferiority of sheathless RIRS in appropriately selected patients. Sixth, this study was performed using a holmium:YAG laser; therefore, our findings may not be directly generalizable to RIRS performed with the newer TFL. Given that TFL has been reported to provide superior dusting efficiency and less retropulsion [18], it may further facilitate sheathless RIRS and potentially improve procedural efficiency and safety, but this hypothesis requires confirmation. Future studies comparing sheathless RIRS outcomes using holmium vs. TFL are warranted.

Our findings indicate that sheathless RIRS is a viable and effective alternative for certain patients, particularly those with minimal stone loads or in cases when UAS implantation fails. This method may prevent superfluous ureteral dilatation and mitigate potential ischemia risks linked to UAS, without jeopardizing surgical results. As laser efficiency improves and ureteroscope diameters decrease, the range of cases suitable for sheathless RIRS will likely expand. Future guidelines should incorporate these technological advances and provide evidence-based recommendations for when UAS can be safely omitted versus when it remains beneficial.

## 5. Conclusions

Sheathless RIRS may be a feasible option in carefully selected patients with a small stone burden (<1 cm) or when initial placement of a 10/12 Fr UAS is unsuccessful despite the successful passage of an 8 Fr semirigid ureteroscope, and the surgeon anticipates completion within 2 h. In this real-world cohort, stone-free and perioperative outcomes were not statistically different between sheathless and UAS-assisted RIRS, suggesting that routine UAS use may not be necessary in such scenarios. However, given the retrospective design, stone-size imbalance, and limited sample size, these data should not be interpreted as procedural equivalence or definitive safety across all RIRS candidates. Larger prospective studies with longer follow-up and more robust adjustment are warranted to confirm patient selection criteria and outcomes.

## Figures and Tables

**Table 1:** Baseline patient characteristics between groups.

	UAS(−) (*n* = 18)	UAS(+) (*n* = 37)	*p* Value
Age, years	57 (24–81)	59 (37–79)	0.733
Body height (m)	1.63 (1.48–1.82)	1.615 (1.43–1.79)	0.290
Body weight (kg)	72.2 (43.8–133.1)	67.2 (40.9–100.0)	0.360
BMI, kg/m^2^	25.3 (18.7–40.4)	25.5 (15.8–37.0)	0.760
Gender (male), *n* (%)	12 (66.7%)	22 (59.5%)	0.600
Stone location, *n* (%)			
Renal pelvic stones	3 (16.7%)	8 (21.6%)	>0.999
Upper calyceal stones	2 (11.1%)	8 (21.6%)	0.470
Middle calyceal stones	3 (16.7%)	13 (35.1%)	0.157
Lower calyceal stones	10 (55.6%)	29 (78.4%)	0.080
Stone size (mm)	12 (6–24)	17 (11–40)	0.001 *

* *p* < 0.05. Values are presented as median (range) unless otherwise indicated. *p*-value of continuous variable by Mann–Whitney U test. *p*-value of discontinuous variable by Chi-Square Test or Fisher’s Exact Test when appropriate. UAS = ureteral access sheath, BMI = body mass index.

**Table 2:** Comparison of operative variables and outcomes between groups.

	UAS(−) (*n* = 18)	UAS(+) (*n* = 37)	*p* Value
VAS score (post-operative 2–3 h)	0 (0–3)	0 (0–6)	0.587
Urethral pain, *n* (%)	5 (27.8%)	9 (24.3%)	>0.999
Flank pain, *n* (%)	3 (16.7%)	6 (16.2%)	>0.999
Hospital stays, days	1 (1–6)	1 (1–7)	0.663
Operation time, min	77 (28–128)	85 (34–278)	0.154
Stone-free, *n* (%)	12 (66.7%)	23 (62.2%)	0.745

Values are presented as median (range) or *n* (%), as appropriate. *p*-value of continuous variable by Mann–Whitney U test. *p*-value of discontinuous variable by Chi-Square Test or Fisher’s Exact Test when appropriate. UAS = ureteral access sheath, VAS = visual analogue scale.

**Table 3:** Logistic regression analysis of stone-free rate.

	Univariable Analysis (Crude)			Multivariable Analysis (Adjusted)		
	Odds Ratio	95% CI	*p*-Value	Odds Ratio	95% CI	*p*-Value
Age	0.959	0.914–1.006	0.089	0.965	0.919–1.014	0.161
Gender						
Female	1					
Male	1.128	0.365–3.483	0.834			
BMI	0.995	0.882–1.123	0.937			
Stone size	0.975	0.910–1.045	0.473	0.981	0.905–1.064	0.645
Lower calyceal stones						
No	1			1		
Yes	0.167	0.033–0.834	0.029 *	0.172	0.032–0.923	0.040 *
Scope caliber (Fr.)						
9	1					
7.5	1.172	0.259–5.306	0.836			
UAS use						
No	1			1		
Yes	0.821	0.2514–2.684	0.745	1.408	0.328–6.401	0.645

* *p* < 0.05. BMI = body mass index, UAS = ureteral access sheath.

## Data Availability

The original contributions of the study are contained within the article, and additional inquiries may be made to the corresponding authors.
